# Abnormalities of Resting State Functional Connectivity Are Related to Sustained Attention Deficits in MS

**DOI:** 10.1371/journal.pone.0042862

**Published:** 2012-08-17

**Authors:** Marisa Loitfelder, Massimo Filippi, Mara Rocca, Paola Valsasina, Stefan Ropele, Margit Jehna, Siegrid Fuchs, Reinhold Schmidt, Christa Neuper, Franz Fazekas, Christian Enzinger

**Affiliations:** 1 Medical University of Graz, Department of Neurology, Graz, Austria; 2 Neuroimaging Research Unit, Institute of Experimental Neurology, Scientific Institute and “Vita-Salute” University, Ospedale San Raffaele, Milan, Italy; 3 Institute of Psychology, Karl Franzens University, Graz, Austria; 4 Department of Radiology, Section of Neuroradiology, Medical University of Graz, Graz, Austria; University Hospital La Paz, Spain

## Abstract

**Objectives:**

Resting state (RS) functional MRI recently identified default network abnormalities related to cognitive impairment in MS. fMRI can also be used to map functional connectivity (FC) while the brain is at rest and not adhered to a specific task. Given the importance of the anterior cingulate cortex (ACC) for higher executive functioning in MS, we here used the ACC as seed-point to test for differences and similarities in RS-FC related to sustained attention between MS patients and controls.

**Design:**

Block-design rest phases of 3 Tesla fMRI data were analyzed to assess RS-FC in 31 patients (10 clinically isolated syndromes, 16 relapsing-remitting, 5 secondary progressive MS) and 31 age- and gender matched healthy controls (HC). Participants underwent extensive cognitive testing.

**Observations:**

In both groups, signal changes in several brain areas demonstrated significant correlation with RS-activity in the ACC. These comprised the posterior cingulate cortex (PCC), insular cortices, the right caudate, right middle temporal gyrus, angular gyri, the right hippocampus, and the cerebellum. Compared to HC, patients showed increased FC between the ACC and the left angular gyrus, left PCC, and right postcentral gyrus. Better cognitive performance in the patients was associated with increased FC to the cerebellum, middle temporal gyrus, occipital pole, and the angular gyrus.

**Conclusion:**

We provide evidence for adaptive changes in RS-FC in MS patients compared to HC in a sustained attention network. These results extend and partly mirror findings of task-related fMRI, suggesting FC may increase our understanding of cognitive dysfunction in MS.

## Introduction

Cognitive dysfunction is increasingly recognized as an important aspect of multiple sclerosis (MS). MS-related cognitive impairment may affect memory, processing speed, attention and concentration [1]. It negatively impacts on the quality of life, in part independently from physical disability [2]. To date, there is no established therapeutic intervention to alleviate symptoms or prevent further cognitive decline in MS, although several strategies have been suggested [3].

In this context, methods to assess potential effects of such interventions on brain function both with high sensitivity and objectivity are needed. Behaviorally, this is notoriously difficult [4], and, clearly falls short of providing insights into the underlying mechanisms of potential therapeutic strategies. In this regard, functional MRI (fMRI) is a valuable and objective tool to assess abnormalities of brain function [5]. Functional reorganization in cognitive domains with progression of MS has been demonstrated recently [6].

However, conventional task-related fMRI studies need to resort to isolated tasks and frequently suffer from flooring and ceiling effects in heterogeneously performing, variably impaired, patient groups. Resting state (RS) fMRI has been proposed to overcome these limitations, as it allows an identification of network abnormalities related to disease [7], [8], without subjecting patients to demanding tasks.

More specifically, functional connectivity (FC) MRI at RS can measure the interdependency of correlated brain regions, detecting FC networks while the brain is at rest and not adhered to a specific task [9]–[12]. While RS-fMRI data acquisition usually implies several minutes of baseline scanning with the subjects lying in the scanner with their eyes closed, Fair et al. [13] proposed selected frames of block-design rest phases to be sufficient to assess RS-FC of the brain. This novel approach allows analyzing existing data sets in patient cohorts that are otherwise immanently hard to access repeatedly, and thus obtain new insights into functional network abnormalities from RS activity.

The Paced Auditory Serial Addition Test (PASAT) as well as the Symbol Digit Modalities Test (SDMT), both parts of the Brief Repeatable Battery of Neuropsychological Tests (BRB-N), are widely used to test cognitive dysfunction and decline over time in MS [7], [14]. Demanding executive control, they require sustained and divided attention, information processing, and working memory [15]. Using fMRI, a widespread fronto-parieto-temporal network including the anterior cingulate cortex (ACC) [16], [17] has been identified for working memory tasks and higher executive functions [18]. In particular, the ACC has been defined as a crucial area for PASAT/SDMT performance [17] in MS.

Using the method proposed by Fair et al. [13] and fMRI, we thus chose the ACC as seed-point to assess differences and similarities between MS patients and healthy controls (HC) in RS-FC within a sustained attention network. Further, we sought to define abnormalities of RS-FC related to PASAT and SDMT performance.

## Materials and Methods

Ethics Statement: The ethics committee on human experimentation of the Medical University of Graz approved the study. Participants gave written informed consent.

### Patients and healthy controls

We reanalysed data from a subset of patients and controls recruited for a previous fMRI study [6], who contributed both fMRI data satisfying criteria for FC analyses (i.e., with movement parameters not exceeding 2 mm in any plane and not showing artefacts) and a complete PASAT/SDMT examination. **Table 1** summarizes the main demographic and clinical characteristics of the entire cohort of study participants and subdivided for different phenotypes in the MS-patients. Significant differences within MS patients have been driven by patients with secondary progressive disease course.

**Table 1:** Demographics, clinical characteristics, conventional MRI parameters and neuropsychological test performance.

	Controls (n = 31)	MS-patients (n = 31)	p	CIS	RRMS	SPMS	p
**Demographics**							
age (years)	32.5**±**8.6 (20–52)	35.2**±**10.7(18–53)	0.401^1^	34.6±10.5	31.3±8.9	49.0±3.2	0.002^3^
education (years)	17.9**±**3.3 (12–26)	14.8**±**3.2 (11–23)	<0.001^2^	14.2±2.6	15.6±3.6	13.4±2.5	0.345^3^
**Clinical variables**							
disease duration (years)	NA	5.5**±**6.9 (0.2–24.0)	NA	0.7±0.3	4.6±4.4	18.0±5.1	0.000^4^
EDSS [median]	NA	2.1**±**2.1 (0.0–7.5) [2.0]	NA	0.6±0.8	1.9±1.2	5.9±1.6	<0.001^3^
**MRI data**							
Normalized brain volume (cm3)	1629.8±91.4	1593.7**±**117.7	0.014^2^	1574.3±174.5	1571.9±107.2	1477.5±834.0	0.339^3^
	(1344.9–1796.8)	(1128.7–1796.8)		(1128.7–1752.5)	(1333.3–1738.4)	(1392.8–1569.8)	
T2-lesion load (cm3)	NA	11.8**±**16.4 (0.2–85.5)	NA	4.2±4.6	14.4±21.2	18.9±5.7	0.174^3^
**Cognitive testing**							
*BRB-N*							
PASAT	52.3±8.9	46.1±10.9	0.010^1^	50.0±9.1	47.4±9.8	34.4±11.7	0.021^3^
Long Term Storage	61.3±6.2	57.8±11.1	0.134^2^	59.9±8.6	60.9±10.1	43.6±8.3	0.004^3^
Consistent Long Term Retrieval	57.8±8.6	52.8±15.1	0.118^2^	53.5±13.4	57.8±13.7	35.4±10.9	0.011^3^
Spatial Recall Test	23.5±4.1	22.5±5.5	0.455^2^	24.3±4.7	21.9±6.1	20.75±4.5	0.440^3^
Symbol Digit Modalities Test	59.6±9.0	48.0±15.2	0.001^2^	53.7±8.5	48.5±16.8	31.5±11.6	0.040^3^
Selective Reminding Test (dr)	11.6±0.7	10.9±1.5	0.061^1^	11.1±1.1	11.3±1.1	9.4±2.7	0.180^4^
Spatial Recall Test (dr)	8.6±1.9	8.2±2.0	0.339^1^	8.7±1.5	8.2±2.2	7.0±2.2	0.475^4^
Word List Generation	24.9±5.7	25.4±8.1	0.801^2^	27.2±11.5	25.6±5.9	20.8±4.8	0.355^3^
*WCST*							
Total Number Correct	66.4±4.3	68.3±9.2	0.416^1^	69.5±11.8	68.4±7.1	63.7±11.8	0.644^3^
Total Number of Errors	10.7±5.2	15.1±13.8	0.135^1^	11.0±3.8	14.0±8.1	34.7±37.7	0.333^4^
Perseverative Responses, n	6.1±3.3	8.3±10.3	0.624^1^	4.8±2.2	7.8±6.5	22.3±28.3	0.247^4^
Perseverative Errors, n	5.9±2.8	8.2±8.5	0.238^1^	5.3±1.4	7.8±5.1	19.67±23.7	0.243^4^
Non-Perseverative Errors, n	4.8±3.0	6.9±5.9	0.191^1^	5.7±3.1	6.1±4.1	15.0±14.2	0.035^3^

Data are given as mean ± SD (range);

1 Mann-Whitney U-Test,

2 two sample t-test,

3 ANOVA,

4 KruskalWallis-Test. Normal distribution tested with K-S Test; NA: not applicable. BRB = Brief Repeatable Battery. WCST = Wisconsin Card Sorting Test.

The cohort consisted of 31 patients from the MS Outpatient Department of Neurology at the Medical University of Graz, comprising 10 patients with a clinically isolated syndrome (CIS) suggestive of MS and brain MRI fulfilling criteria of dissemination in space [19], 16 patients with a relapsing remitting and 5 patients with a secondary progressive course of MS [19]. Inclusion criteria were right-handedness [20], clinically preserved function of the right hand, sufficient visual function to recognise the stimuli and behavioural tests, and sufficient cognitive abilities to comprehend procedures. Exclusion criteria were contraindication to MRI, known psychiatric disorders, clinically significant depression or fatigue, and acute relapse or steroid medication four weeks prior to the study. Thirty-one right-handed [20] healthy subjects, unremarkable for neuropsychiatric disorders and with a normal neurological examination, served as age- and gender-matched controls (HC).

### Clinical and neuropsychological assessment

A single neurologist assessed patients' disability using the EDSS [21] at the day of the neuropsychological assessment. Subjects were tested by trained psychologists, blinded to clinical and paraclinical data, one to seven days prior to scanning. The test battery included the BRB-N [22], and the Wisconsin Card Sorting Test (WCST) [23] to assess higher executive abilities [6].

### MRI data acquisition

Imaging was performed at 3.0 T (Tim Trio, Siemens, Erlangen, Germany) using a 12-element head coil. A single shot gradient-echo EPI-sequence (TR = 3000 ms, TE = 30 ms, FA = 90°, matrix size = 64×64, pixel size = 3.0×3.0 mm^2^) was used for fMRI, discarding the first two volumes to account for T1-saturation. The total number of volumes was 192, each comprising 36 slices with 3 mm thickness. A high-resolution T1-scan served for functional image registration to precisely locate activations (3D-MPRAGE; TR = 1900 ms, TE = 2.6 ms, TI = 1900 ms; 1×1×1 mm^3^ resolution) and to calculate normalized brain volume (NBV) using SIENAx (part of FSL, FMRIB's Software Library, www.fmrib.ox.ac.uk/fsl). T2-hyperintense lesion load (LL) was computed on FLAIR sequences (TR = 10000, TE = 69, TI = 2500, flip angle = 160, 44 slices, thickness = 3 mm) by a blinded trained assessor using DispImage software, after lesion identification by an experienced rater, as described previously [6].

### fMRI data analysis

During the fMRI experiment [6], subjects had to perform a stimulus-response discrimination (Go-No Go) task, including aspects of inhibition/disinhibition, in a block design (ABAB), where 10 active runs (A) were intermittently presented with 9 rest phases (B). Active phases lasted 10 volumes (30 s), rest phases 7 volumes (21 s) each. Prior to each active phase, an instruction and a countdown, each lasting one volume, were presented. One of two pseudo-randomized presented stimuli constituted the target to which subjects had to respond as fast as possible [6].

To assess FC between functionally related brain regions, we performed correlation analyses between the pre-specified seed region and all remaining voxels. We processed fMRI time series as follows: first, data were pre-processed using statistical parametric mapping (SPM5) software (Wellcome Department of Cognitive Neurology). This included realignment and normalization to the standard Montreal Neurological Institute space. Then, the software REST 1.3 (Song Xiaowei, http://resting-fmri.sourceforge.net) was used for the additional pre-processing steps aiming to reducing spurious variance. These included: 1) band-pass filtering (0.009 Hz<f<0.08 Hz); 2) spatial smoothing (using SPM5) with a 6×6×6 mm full-width at half maximum (FWHM) Gaussian filter, 3) regression of the six parameters obtained by rigid body head motion correction; 4) regression of the whole brain signal averaged over the whole brain; 5) regression of ventricular signal averaged from a ventricular region-of-interest (ROI); and 6) regression of white matter (WM) signal averaged from a WM ROI [13].

After pre-processing, we selected functional data from the relative rest periods only for further analyses according to the criterion by Fair et al. [13]: (1) two volumes after the start of each task block were included (∼6 s) to account for the hemodynamic delay, and (2) at the end of each task block 5 (∼15 s) volumes were excluded from RS data, allowing the hemodynamic response to return to baseline. This procedure provided 45 volumes for FC-analyses for each subject.

FC-maps were then calculated based on an *a priori* defined cluster mask of the ACC, created with the WFU-Pickatlas-toolbox [24]. This seed region was chosen as it describes a crucial component within the attention system [16], [25]. FC was calculated assessing the correlation coefficient between the average time series of the ACC and any other voxel of the brain. A Fisher's-z transformation was applied to improve the Gaussianity of the obtained correlation coefficients. This allowed the individual creation of a RS-FC map with the ACC.

### Statistical analysis

Individual FC spatial maps of z-scores were entered in a SPM5 random-effect analysis to assess the main RS-FC with the ACC in controls and patients separately (one-sample t-test, p<0.05, family-wise error [FWE] corrected for multiple comparisons). A two-sample t-test served to assess between-group differences of RS-FC between controls and patients, whereas multiple regression models were used to assess correlations between RS-FC and PASAT/SDMT performance (p<0.001, uncorrected for multiple comparisons). Additional multiple regression analyses were conducted with clinical data and T2-lesion load. For all analysis, only clusters exceeding a size of k = 30 voxels are reported.

The Statistical Package of Social Sciences (17.0; SPSS Inc., Chicago, IL) was used to test inter-group differences.

## Results

**Table 1** summarizes the main demographic, clinical and structural MRI data. Patients and HC did not differ regarding age, but patients had experienced fewer years of education. Disability of MS patients was low on average, but spanned a wide range. As expected, NBV was lower in patients compared with controls. Regarding cognitive functioning, patients solely performed worse than controls in the PASAT (p = 0.010) and SDMT (p = 0.001). Differences in the PASAT were driven by 7 patients (4 SPMS, 3 RRMS) and in the SDMT by 6 patients (3 SPMS, 3 RRMS; four persons overlapping with the former group), who performed one standard deviation worse than the group mean of the patients. This performance was also one standard deviation worse than the group mean of the controls. There was no significant difference in WCST performance.

### FC at group level and differences between patients and controls

**Figure 1:**
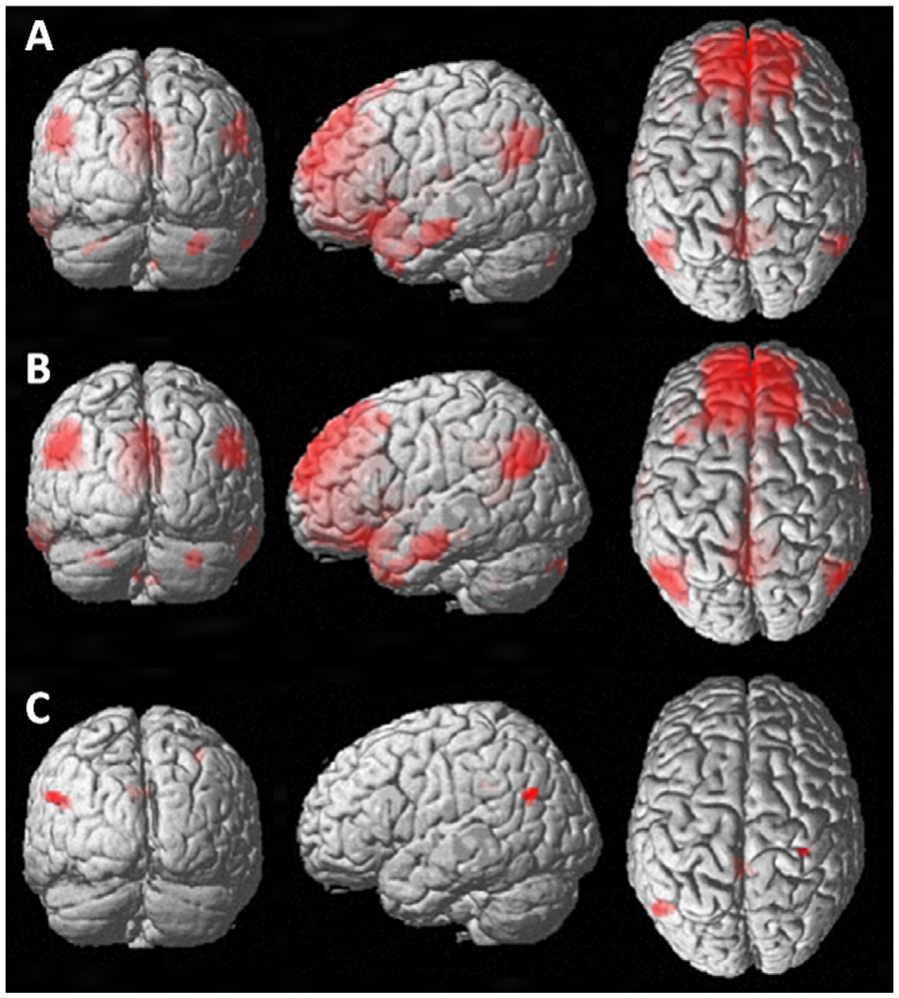
Functional connectivity (FC) in relation to the anterior cingulate cortex (ACC) in healthy controls (A) and MS patients (B) in periods of relative rest. MS patients (compared to controls) demonstrated increased FC to the right postcentral gyrus, the left angular gyrus and the left posterior cingulated cortex (C) (Images shown in coronal, sagittal, and axial orientation in neurological convention).

In both patients and HC, several brain areas demonstrated significant RS-FC with the ACC (**Figures 1A and B**). These included the posterior cingulate cortex (PCC), insular cortices, the right (R) caudate, R middle temporal gyrus, angular gyri, the R hippocampus, and R cerebellum (crus 1 and 2).

Additionally, RS-FC with the ACC was detected for the left (L) precuneus, L medial cingulate cortex, and R cerebellum (crus 2) in HC; and, in patients, for the parahippocampal gyri, L paracingulate gyrus and bilateral cerebellum (lobule IX, crus 1) (**Table 2**).

**Table 2:** Average resting state (RS) functional connectivity (FC) with the anterior cingulated cortex (ACC) in controls and MS patients (one-sample t test, p<0.05 FWE corrected) and differences in RS-FC between these groups (two-sample t test, p<0.001, cluster extent k = 30).

	*x*	*y*	*z*	*kE*	*pcorr*	*area*	*Side*
**Healthy controls (HC)** 1	6	40	20	13788	0.000	ACC	R
	32	16	−18	4657	0.000	Insula, frontal orbital cortex	R
	16	16	12			Caudate	R
	−30	14	−18			Insula, frontal orbital cortex	L
	0	−54	32	2326	0.000	PCC, precuneus	L
	0	−18	36	340	0.000	Medial cingulate cortex	L
	48	4	−40	356	0.000	Middle/inferior temporal gyrus	R
	62	−10	−18	127	0.009	Middle temporal gyrus	R
	−50	−62	28	721	0.000	Angular gyurs	L
	26	−16	−22	104	0.024	Hippocampus	R
	30	−82	−36	146	0.004	cerebellum Crus 2	R
	34	−86	−30			cerebellum Crus 1	R
	58	−60	38	337	0.000	Angular gyrus	R
**MS patients** 1	0	48	8	19229	0.000	ACC, paracingulate gyrus	L
	−2	58	14			Frontal pole	L
	−52	−66	28	1160	0.000	Angular gyrus	L
	−60	−10	−20	1802	0.000	Middle temporal gyrus	L
	−28	14	−16			Insula	L
	30	18	−20	2257	0.000	Insula	R
	34	30	−22			Frontal orbital cortex	R
	16	18	12			Caudate	R
	54	−66	28	607	0.000	Angular gyrus	R
	−24	−20	−22	359	0.000	Parahippocampal gyrus	L
	30	−16	−24	203	0.001	Parahippocampal gyrus	R
	24	−10	−22			Hippocampus	R
	34	−26	−22			Hippocampus/Fusiform gyrus	R
	8	−50	−46	101	0.047	cerebellum IX	R
	−4	−54	−42			cerebellum IX	L
	32	−86	−34	111	0.031	cerebellum Crus 1	R
**MS>HC** 2	34	−32	52	33	0.796	Postcentral gyrus	R
	−52	−66	32	71	0.210	Angular gyrus	L
	−6	−38	32	68	0.236	PCC	L
**Correlation analyses**							
**HC: better PASAT performance**	−10	0	12	61	0.192	Caudate	L
	−40	−54	42	81	0.071	Angular gyrus	L
**HC: worse PASAT performance**	4	58	−16	99	0.030	Frontal Pole	R
	8	58	38	44	0.441	Frontal Pole	R
	26	44	50	33	0.697	Superior frontal gyrus	R
	−46	−6	−32	31	0.746	Inferior temporal gyrus	L
	60	−2	−24	30	0.770	Middle temporal gyrus	R
	−54	22	34	44	0.441	Middle frontal gyrus	L
	−8	−24	30	31	0.746	Medial cingulated cortex/PCC	L
	54	34	30	42	0.483	Middle frontal gyrus	R
	6	−22	−40	60	0.266	Brain stem	R
**HC: better SDMT performance**	−14	−40	72	43	0.450	Postcentral gyrus	L
	38	−30	−12	33	0.688	Hippocampus	R
**HC: worse SDMT performance**	10	56	−6	68	0.129	Orbitofrontal cortex	R
	2	64	−10				R
**MS: better PASAT performance**	−24	−40	−34	178	0.002	Cerebellum	L
	−46	−50	−28			Cerebellum crus 1	L
	−36	−48	−30			Cerebellum VI	L
	36	−54	−38	37	0.668	Cerebellum crus 1	R
	−56	−8	−22	31	0.798	Middle temporal gyrus	L
	−18	−102	−18	32	0.778	Occipital pole	L
	−44	−64	30	36	0.690	Angular gyrus	L
**MS: worse PASAT performance**	62	14	8	33	0.756	Inferior frontal gyrus, pars opercularis	R
	20	14	16	33	0.756	Caudate	R
**MS: better SDMT performance**	22	−82	−38	98	0.049	Cerebellum crus 2	R
	8	−60	−48	80	0.108	Cerebellum 9	R
	−52	−62	46	37	0.665	Parietal inferior cortex	L
	42	−68	−42	40	0.599	Cerebellum crus 2	R
**MS: worse SDMT performance**	26	4	2	71	0.161	Putamen	R
	30	−4	2			Putamen	R
	38	8	−4	30	0.817	Putamen	R
	30	14	−6			Putamen	R
**T2LL (positive)**	−4	−74	−20	31	0.799	Cerebellum 6	L
**EDSS (negative)**	−26	−50	−30	277	0.000	Cerebellum 6	L
	−14	−58	−16			Cerebellum 6	L
	−20	−52	−16			Cerebellum 4/5	L
	34	−52	−26	174	0.000	Cerebellum 6	R
	30	−42	−24			Cerebellum 4/5	R
	26	−60	−28			Cerebellum 6	R
	20	−24	−20	39	0.031	Parahippocampal gyrus	R
	−18	−96	−18	37	0.035	Lingual gyrus	L
**DD (negative)**	−58	−12	−26	37	0.666	Temporal inferior	L
	44	−70	−12	38	0.664	Occipital inferior	R
	−20	−62	−22	115	0.024	Cerebellum 6	L
	−40	−48	−30			Cerebellum crus 1	L
	−26	−52	−30			Cerebellum 6	L
	28	−58	−30	51	0.389	Cerebellum 6	R
	24	−62	−20			Cerebellum 6	R
	−50	−64	46	37	0.035	Angular gyrus	L

Abbreviations: A/PCC = anterior/posterior cingulated cortex, T2LL = T2 lesion load, DD = disease duration;

1 corrected (FWE, p<0.05),

2 uncorrected (p<0.001).

Between-group statistical comparisons revealed increased RS-FC between the ACC and the R postcentral gyrus, the L angular gyrus, and the L PCC in patients vs. HC (**Figure 1C** **,** **Table 2**).

### FC and PASAT/SDMT performance

**Figure 2:**
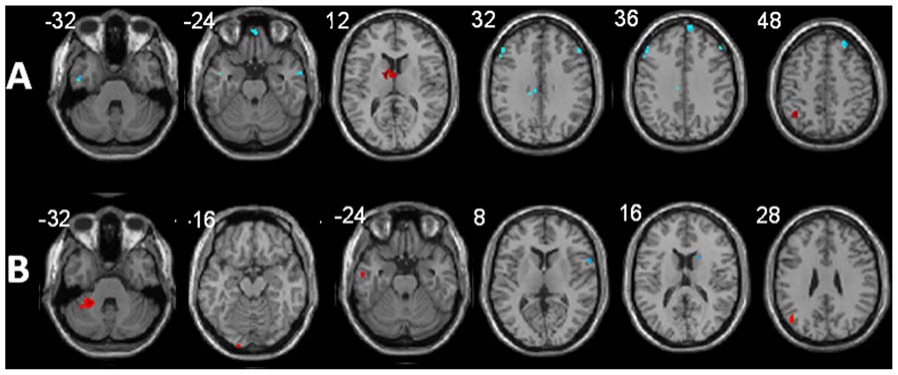
Modulation of functional connectivity (FC) in relation to the anterior cingulate cortex (ACC) by PASAT performance in healthy controls (A) and MS patients (B). Areas demonstrating increased FC with better performance are shown in red, areas demonstrating increased FC with worse performance are shown in blue (selected axial slices in neurological convention). For further explanations please see text.

Given an identical range of PASAT performance in MS patients and HC (23–60), we also sought to specifically assess the relationship between FC of regions belonging to this cognitive network and behavioral performance, separately for HC and patients. Results of multiple regression models revealed in HC a positive correlation between PASAT performance and RS-FC to the L caudate nucleus and L angular gyrus; while PASAT performance negatively correlated with RS-FC to the R frontal pole, R superior frontal gyrus, L inferior and R middle temporal gyrus, R middle frontal gyrus and L medial cingulate cortex (**Figure 2A**).

In MS patients, PASAT performance positively correlated with RS-FC to the L cerebellum (lobule VI, crus 1), R cerebellum (crus 1), L middle temporal gyrus, L occipital pole, and – as in HC – the L angular gyrus. Patients showed a negative correlation between PASAT performance and FC to the R pars opercularis of the R inferior frontal gyrus and the R head of the caudate nucleus (**Figure 2B**).

**Figure 3:**
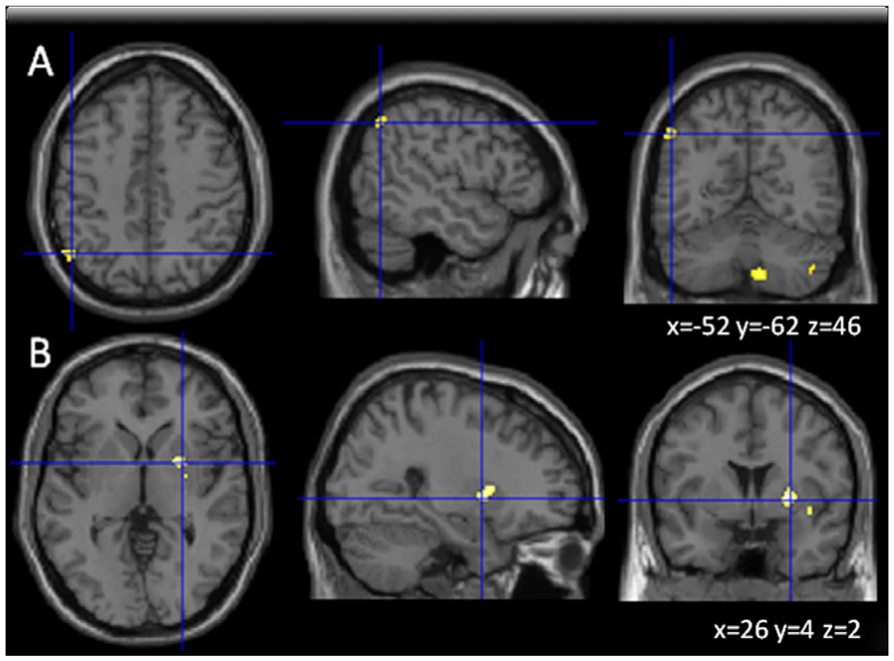
Brain areas showing positive (A) and (B) negative correlation between functional connectivity in relation to the anterior cingulated cortex (ACC) and Symbol Digit Modalities Test performance (SDMT, part of the BRB-N) in MS patients. (Images shown in axial, sagittal and coronal orientation in neurological convention).

We also performed analogue FC-analyses for the SDMT. These analyses yielded positive correlation for FC in MS patients to the L parietal inferior cortex and R cerebellar regions (crus 2, crus 9) with SDMT performance (**Figure 3A**) and negative correlation of the R putamen with SDMT performance (**Figure 3B**).

### Correlation of FC with clinical and other MRI parameters

We also tested whether clinical parameters and T2-LL have an influence on RS-FC. EDSS severity and disease duration correlated inversely with RS-FC of the ACC with bilateral cerebellar areas, especially area 6. Disease duration also correlated inversely with RS-FC with the angular gyrus. T2-LL correlated with FC to cerebellar region 6 (**Table 2**).

## Discussion

The aim of this study was to test for differences and similarities of RS-FC in a network relevant for sustained attention in MS patients *vs*. HC, using a novel approach to analyze resting state data [13] from existing block-design fMRI data [6]. To achieve this goal, we chose the ACC as the seed region, as it has been identified as a crucial component of the cognitive network active during PASAT performance, a sustained attention demanding task [16], [18], [25]–[27]. The PASAT also requires working memory, information processing and executive control for task maintenance, thus involving many cognitive processes that may be impaired in MS [28].

As a primary finding, our seed-region based FC-analyses identified virtually the same attention network components that have been reported in task-related fMRI [29], [30]. This applied to both patients and HC, attesting the validity of the approach used to capture brain areas involved in cognitive function that are to some degree functionally connected [31].

As a secondary major finding, direct comparison of RS-FC with the ACC revealed differences between patients and HC. These included increased FC in the patients to the postcentral and angular gyri, and the PCC. No area showed increased FC in controls. In the patients group, specific cerebellar regions also showed FC to the ACC, although differences were not statistically significant when compared to controls. It has been previously suggested that areas of the cerebellar cortex are interconnected with prefrontal regions and therefore critical in the execution of cognitive tasks [32], [33]. However, analyses also demonstrated decreased FC with increasing EDSS and longer disease duration, suggesting that with disease progression, connectivity to the cerebellum might get increasingly lost in specific circumscribed cerebellar areas. This implies that reorganization within the cerebellar system to maintain cognitive performance in MS is certainly complex, may be limited at some point, and not equally effective in each cerebellar portion. However, previous conventional fMRI activation studies provided evidence for functional cortical reorganization during PASAT performance already in the early stages of MS [29], [30], [32], [33], which is consistent with our findings. Our results also suggest that widespread dysfunction of the brain in a functional network related to attention is also present in MS patients at relative rest.

As a third research question, using multiple regression analyses, we sought to assess whether RS-FC correlated with actual behavioral function, as assessed by the PASAT and the SDMT. We found some differences and similarities between patients and HC regarding the relationship between FC patterns and PASAT/SDMT performance. Given the fact that HC showed the same range of PASAT performance as patients (although, on average, patients performed worse), we deemed necessary to also assess these correlations in the group of HC. Our results point to the L angular gyrus and the parietal inferior cortex as regions of particular interest, as a correlation between FC to the ACC and PASAT/SDMT performance was evident. Task-related fMRI already demonstrated angular gyrus activation to be crucial for the Paced Visual Serial Addition Test (PVSAT) in MS, which has been interpreted as compensatory mechanism [16]. Our observation of the positive correlations of FC with these regions and PASAT/SDMT performance also in HC extends these findings, allowing the conclusion that angular gyrus/parietal inferior cortex activation might be important for performance in sustained attention tasks. Together, this underscores the importance of these regions in cognitive processes. Increased FC to these areas in MS patients suggests that FC may gain additional importance in the quest to functionally adapt to MS related pathology. Partially in line with this notion, activation shifts towards the angular gyrus and more frontal areas [16], [25] were noted in relapsing-remitting MS patients.

In addition to the angular gyrus, we found a positive correlation between FC of the ACC with the L caudate nucleus and PASAT performance in HC, whereas, somewhat suprisingly, the opposite trend was observed in MS patients. Further, there was negative correlation between FC of the ACC with SDMT performance and the putamen. These results suggest that RS activity in the head of the caudate nucleus as well as the putamen, i.e., the dorsal striatum, has important relation with subjects' cognitive performance. Alterations in or disruption of critical cortico-subcortical white matter tracts might explain the negative correlation between cognitive performance and RS-FC in MS patients, but this hypothesis needs to be confirmed by a more comprehensive assessment including a combined assessment of diffusion tensor imaging (DTI) [25] and FC changes.

In contrast to HC, patients exhibited increased FC with better PASAT performance to the cerebellum, the middle temporal gyrus, occipital pole and angular gyrus. As mentioned earlier, there is increasing appreciation that the cerebellum not only plays an important role in the control of movements, but also in attention, problem solving, sensory acquisition, and timing [26], [34].The importance of cerebellar function during PASAT performance has also been emphasized in HC [35], where reciprocal projections from prefrontal cortex to vermal territories of the cerebellar cortex within lobule VII have been identified. Additionally, significant activation in the dorsal, ventral and medial cerebellar cortex has been observed during PASAT performance [35]. Because of the importance of an intact cerebellar function in performing cognitive tasks, we interpret the increased FC to the identified cerebellar regions and the angular gyrus in the MS patients as evidence of adaptive changes.

Several limitations have to be considered in the interpretation of our results. One major disadvantage of the method of RS data analysis used here includes the necessity to define an a-priori hypothesis regarding the seed region for the extraction of the BOLD time-courses to determine the temporal correlation between signal changes in voxels. While the groundings for choosing the ACC as seed region seem solid given the importance of this region for the attention system [16], [25], this approach also limits the identification of further brain regions relevant for working memory. A way to overcome this might have been the use of independent component analysis (ICA), which, however, bears the problem of subjective component selection. Regarding methodology, it needs to be emphasised that our study differs from conventional resting state fMRI studies, as we here extracted specific frames of a conventional block-designed fMRI experiment following certain criteria, rather than obtaining resting state data only over several minutes. Therefore, a residual effect of the active conditions on the defined frames of relative rest cannot be fully excluded. Moreover, a validation of this methodological concept in MS patients is still missing so far. For this, a direct comparison of conventional resting state data and resting state data extracted from a conventional fMRI block designed study obtained in the same patients at very close time-points would have to be conducted. We hope our study stimulates such future research to settle this important issue. First evidence has already been given by Fair et al. They pointed out that such an approach yielded only minor differences with respect to conventionally acquired continuous resting state data [13]. An additional methodological issue that needs to be considered refers to the fact that the data in our analyses are uncorrected for multiple comparisons. However, we chose a p-value of <0.001 and a minimum cluster extent of >30 voxels, similar to the approach that has been used for explorative questions by others [36]–[38]. These values have been chosen based on the fact that the likelihood of two or more voxels exceeding the threshold and to additionally be contiguous is lower than the simple probability of exceeding threshold [39]. Another critical aspect is over-education in the controls compared to the patients, as often noted in clinical populations. However, we are not aware of studies addressing the issue of how educational status affects RS activity, although a recent fMRI study suggested lifetime intellectual enrichment to be linked with cerebral efficiency in MS [40]. Taken together, it appears unlikely that this aspect fully accounts for the key findings of our study.

## Conclusions

In summary, while RS FC-analyses clearly cannot fully replace conventional fMRI studies, our data demonstrate that they provide useful complementary information (e.g., compare to [6]). FC-analyses may also prove to be particularly useful in more impaired patients who cannot accomplish complex tasks or to circumvent flooring and ceiling effects often encountered with cognitive testing.
